# GMSRI: A Texture-Based Martian Surface Rock Image Dataset

**DOI:** 10.3390/s21165410

**Published:** 2021-08-10

**Authors:** Cong Wang, Zian Zhang, Yongqiang Zhang, Rui Tian, Mingli Ding

**Affiliations:** 1 School of Instrumentation Science and Engineering, Harbin Institute of Technology, Harbin 150001, China; 18b901007@stu.hit.edu.cn (C.W.); 20b901007@stu.hit.edu.cn (Z.Z.); tianrui18845159152@163.com (R.T.); 2 Shanghai Institute of Satellite Engineering, Shanghai 200240, China

**Keywords:** Mars image dataset, Martian surface rock image, generative adversarial network

## Abstract

CNN-based Martian rock image processing has attracted much attention in Mars missions lately, since it can help planetary rover autonomously recognize and collect high value science targets. However, due to the difficulty of Martian rock image acquisition, the accuracy of the processing model is affected. In this paper, we introduce a new dataset called “GMSRI” that is a mixture of real Mars images and synthetic counterparts which are generated by GAN. GMSRI aims to provide a set of Martian rock images sorted by the texture and spatial structure of rocks. This paper offers a detailed analysis of GMSRI in its current state: Five sub-trees with 28 leaf nodes and 30,000 images in total. We show that GMSRI is much larger in scale and diversity than the current same kinds of datasets. Constructing such a database is a challenging task, and we describe the data collection, selection and generation processes carefully in this paper. Moreover, we evaluate the effectiveness of the GMSRI by an image super-resolution task. We hope that the scale, diversity and hierarchical structure of GMSRI can offer opportunities to researchers in the Mars exploration community and beyond.

## 1. Introduction

In 2012, the NASA Mars Science Laboratory (MSL) Curiosity rover landed on Mars and began its Mars exploration mission. The Curiosity rover is designed to assess whether Mars ever had an environment to support small life called microbes. In other words, its mission is to determine the habitability of Mars.

Rocks are one of the main substances of the Martian crust, and their texture and shape could provide rich information for planetary geology research [1,2,3,4]. In order to realize the function of rock analysis, a Navigation camera (Navcam) and a Mast camera (Mastcam) are equipped in the Curiosity rover to help the rover to plan routes and collect samples separately [5,6,7,8,9,10]. In the era of artificial intelligence, image-based machine learning is indispensable in assisting the rover to process data from cameras [11,12,13,14]. More sophisticated and robust models and algorithms can be proposed by exploiting extensive images, resulting in better applications for the rover to detect Martian rocks [15,16].

However, when it comes to the problem of getting a lot of Martian rock images, exactly how such a database can be provided is a problem yet to be solved [11,17]. In this paper, we introduce a new image database called “GMSRI”, which provides numerous Martian rock images. We believe the database is a critical resource for developing advanced and large-scale content-based Martian rock image processing algorithms, as well as for providing critical training and bench-marking data for such algorithms.

GMSRI uses a hierarchical structure to organize the real and the generated Mars images. The real Martian rock images are selected from mars32k dataset, which is a public dataset that contains images of various geographical and geological features of Mars, such as mountains and valleys, craters, dunes and rocky terrain. All the images in mars32k are collected by the Mastcam of the Curiosity rover; about 32,000 color images were shot on Mars between August 2012 and November 2018. All images have been scaled-down using linear interpolation to 560 × 500 pixels (some images have been cropped).

In GMSRI, we classify images in terms of both texture and spatial structure. We aim to provide on average 3000–12,000 images to illustrate each category. In order to solve the problem of insufficient data, a state-of-the-art GAN has been trained for producing realistic new images. In this paper, we report the current version of GMSRI, consisting of five sub-trees: Igneous rocks, sedimentary rocks, cracked rocks, gravels, sands. These sub-trees contain 28 leaf nodes and 30,000 images. To sum up, this paper makes the following three main contributions:

(1) A new Martian surface rock image dataset, termed GMSRI, is built to solve the problem of lack of enough data when designing the algorithms for the visual tasks of the Martian rover. GMSRI makes it possible to design more robust and sophisticated models.
(2) A style-based GAN structure is used to fit the distribution of Martian surface rock images and generate images for expanding the dataset, where the synchronously trained discriminator network makes the fitting process of the generator network smoother, and the latent space mapping network and the style transfer network enable us to generate more diverse images in a controllable way.
(3) Experiments are conducted on the task of Mars image super-resolution to verify the effectiveness of the built GMSRI dataset, we achieve 26.42/0.72 and 25.74/0.628 in PSNR/SSIM with ×2 and ×4 scales, respectively, which is a baseline for comparison by other researchers in the future.

The rest of the paper is organized as follows: We first show that GMSRI is a large-scale and diverse image database in Section 1. In Section 2, we describes how GMSRI is constructed by selecting images from relevant datasets and generating images to further expand the dataset. Section 3 presents a simple application example by exploiting the current GMSRI. Our goal is to show that GMSRI can serve as a useful resource for visual recognition applications such as image super-resolution, classification, and detection, etc. This is followed by the conclusions in Section 4.

## 2. Properties of GMSRI

GMSRI is built upon a hierarchical structure. In its completion, GMSRI aims to contain approximately 30,000 images of Martian rocks. As mentioned before, GMSRI consists of five sub-trees: Igneous rocks, sedimentary rocks, cracked rocks, gravels, sands. In the following parts, we give detailed descriptions of the properties of GMSRI.

**Source.** The images of GMSRI come from two sources: The real Martian rock images and the synthesized Martian rock images. The real Martian rock images of GMSRI are selected from mars32K, which is an unlabeled dataset consisting of 32,368 color images collected by the Curiosity rover on Mars between August 2012 and November 2018. The images show various geographical and geological features of Mars such as mountains and valleys, craters, dunes and rocky terrain. All images have been scaled down using linear interpolation to 560 × 500 pixels. The images of mars32K are acquired by the Mastcam of Curiosity, which consists of a pair of focusable digital CCD (Charge Coupied Device) cameras, that can acquire multi-spectral (400–1000 nm) images of the Martian surface and atmosphere at two specific fixed focal lengths [7]. The cameras are mounted atop a 2 m tall mast that enables them to be rotated 360${}^{\circ }$ in azimuth and ±90 ${}^{\circ }$ in elevation. The left Mastcam (M34) has a fixed focal length of 34 mm and a 15 ${}^{\circ }$ field of view, while the right Mastcam (M100) has a fixed focal length of 100 mm and field of view of 5 ${}^{\circ }$ [7]. The left camera’s field trebles that of the right one [8], and the right camera has three times better resolution than that of the left camera. As for the synthesized Martian rock images, the following section will give a detailed introduction and its characteristics.

**Figure 1:**
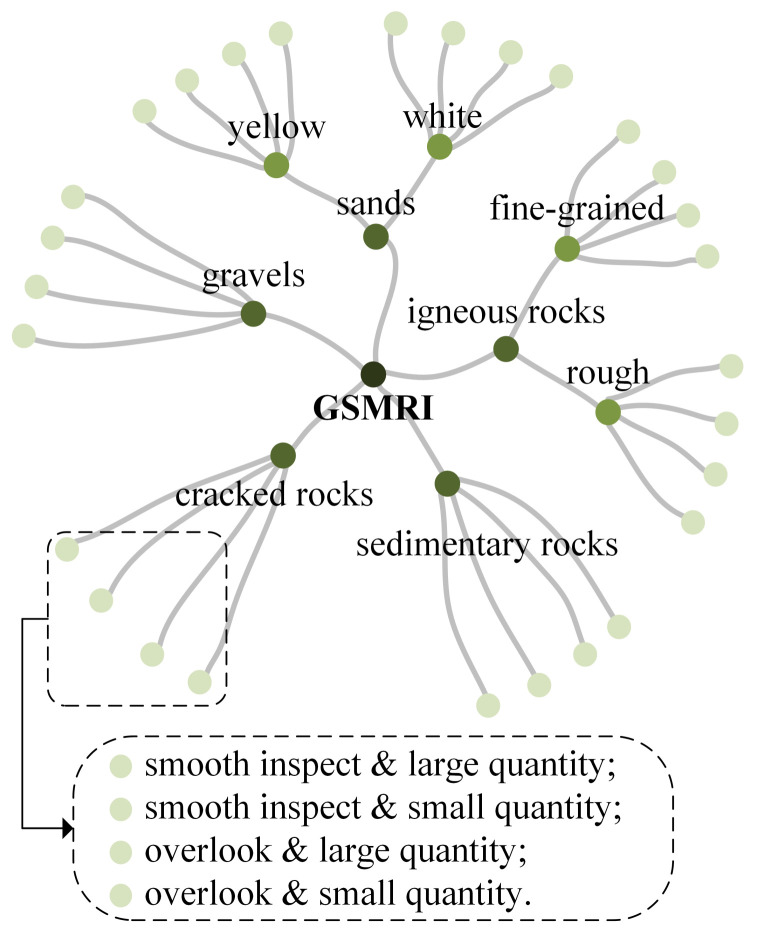
The hierarchy of GMSRI. GMSRI is a four-level tree structure, the second level corresponds to five rock categories, the third level contacts subdivide some rock categories, and the fourth level nodes classify rocks from view angle and quantity of rocks.

**Hierarchy.** We expect that GMSRI organizes the different classes of Martian rock images in a semantic hierarchy. However, the images in mars32K are unlabeled. To overcome this issue, we adopt the tree structure to build the GMSRI dataset. In the tree structure of GMSRI, we divide the Martian rocks by texture into five categories—igneous rocks, sedimentary rocks, cracked rocks, gravels and sands—and create five corresponding sub-trees. The image is then subdivided into 28 leaf nodes based on texture and shape. The hierarchy of GMSRI is shown in Figure 1. From Figure 1, we can observe that GMSRI provides a dense tree structure.

**Figure 2:**
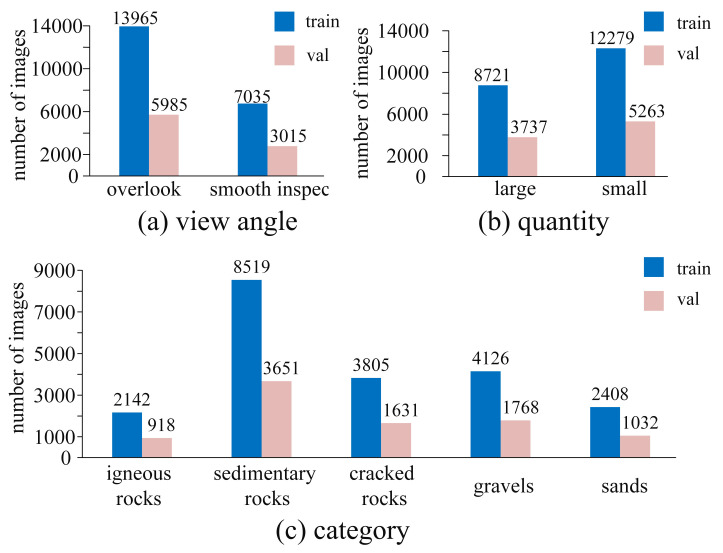
The marginal distributions of the number of images in the current GMSRI. (**a**) Number of images in each view angle. (**b**) Number of images in each quantity. (**c**) Number of images in each category.

**Scale.** GMSRI aims to provide a comprehensive and diverse coverage of the Martian rock images. The current five categories, namely igneous rocks, sedimentary rocks, cracked rocks, gravels and sands, consist of a total of 30,000 images, all of which are 560 × 500 pixels and are divided into training sets and test sets in a ratio of 7 to 3. In addition to the texture-based rock category labeling, we also label the images from the view angle and the quantity of rocks. Figure 2 shows several marginal distributions of the number of images in the current GMSRI.

**Figure 3:**
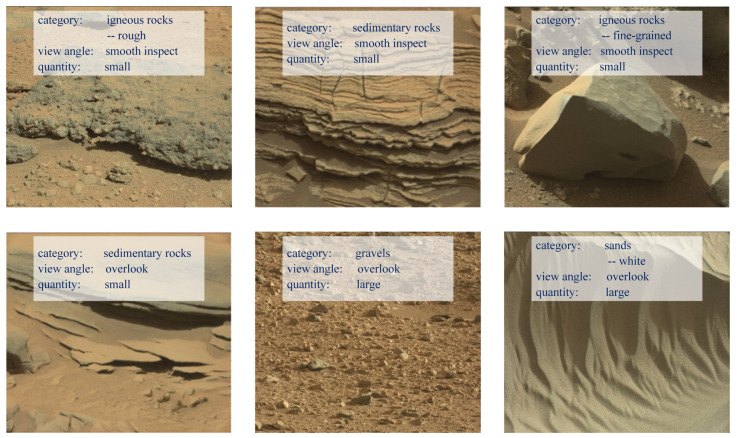
Examples of various spatial structures in our dataset. GMSRI includes a diverse set of 30,000 Martian rock images under different view angles and quantities.

**Diversity.** GMSRI is constructed with the goal that rocks in images should have variable textures and structures, so we constructed the hierarchy of GMSRI from these two aspects. Observing the images in GMSRI, we can see that the differences between the images are not only in the categories of rocks, but also in the quantity of rocks and the view angle from which they are taken. We extracted several images from GMSRI to show the diversity of this dataset, as shown in Figure 3. The diversity ensures that GMSRI has a good generalization ability.

## 3. Building GMSRI

GMSRI aims at providing extensive Martian rock images with their label, that label is used to describe the texture and spatial structure of rock images. In this section, We describe the method used to construct GMSRI.

### 3.1. Overview

**Figure 4:**
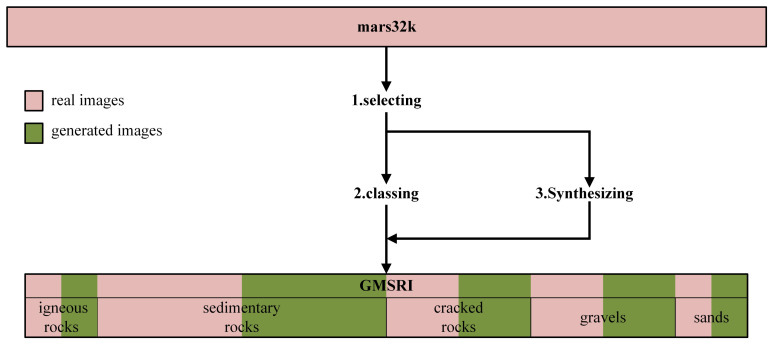
Overview of our proposed method. Mars32k is the database of raw Mastcam images. The processing steps are as follows: 1. Small-field rock images are selected from mars32k. 2. Small-field rock images are classed into five subsets. 3. Using a style-based generator, which is trained by selected images, to synthesis different types of Martian rock images. GMSRI is made up of the selected real images and the generated images.

The first stage of the construction of GMSRI is to collect candidate images. In this paper, 19,687 small-field rock images were selected from mars32k as real candidate images. Secondly, we create a semantic hierarchy from both the texture and shape of the rock data. Then, we use real candidate images selected from mars32k dataset to train a style-based generator [18], which is further used to generate Martian rock images with a technique named style mixing. Finally, the set of each synset is padded by the real candidate images and the synthesized images, and the dataset is expanded to 30,000 images. The overall architecture of our method is shown in Figure 4.

### 3.2. Selecting and Classing Images

**Figure 5:**
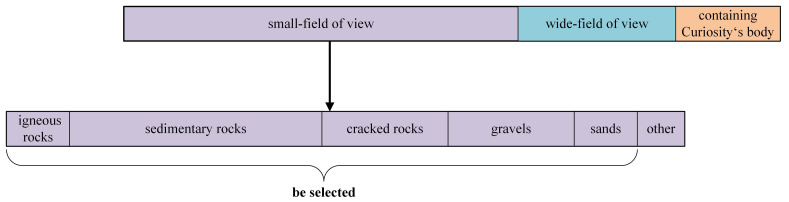
Composition of mars32k. Mars32k contains 32,368 images, including 19,867 images with small-field, 7950 images with wide-field and 4731 images with Curiosity‘s body. The small-field images include 1530 igneous rock images, 5954 sedimentary rock images, 2718 cracked rock images, 2947 gravel images, 1720 sands images and 4998 unclassified images.

**Figure 6:**
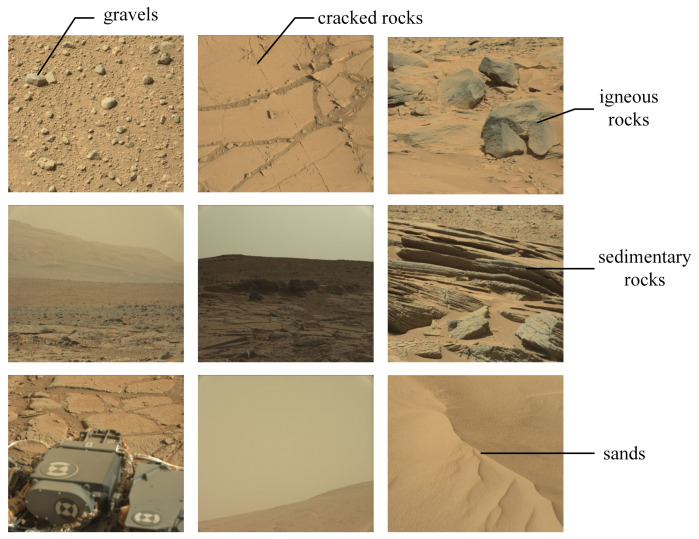
Snapshots of mars32k. We exhibit five representative small-field images, three wide-field images and one image with Curiosity‘s body.

Mars32k consists of about 32,368 color images collected by the Curiosity rover on Mars. The dataset can be broadly divided into small-field of view, wide-field of view and images containing Curiosity‘s body. The small-field images tend to focus on the rocks and contain more textural details, so we selected rock images from the small-field images and classed these into five subsets, namely igneous rocks, sedimentary rocks, cracked rocks, gravels and sands. Composition of mars32k is shown in Figure 5. Igneous rocks mainly include basalt and intrusive rocks [11]. The sedimentary rocks in mars32k are mostly layered sedimentary rocks [19]. Cracked rocks is rocks that have broken into many pieces. Gravels is the smaller rocks. There are also sands on Mars, and we incorporated that into GMSRI. Snapshots of the mars32k are shown in Figure 6.

### 3.3. Generating Images

After the selection stage, we got about 15,000 Martian rock images. However, deep learning based methods are always data hungry if the amount of data can be increased and the trained model based on GMSRI will have higher performance [11,20,21]. Due to the difficulty of Martian rock image acquisition, we need to augment images. Traditional image augmentation methods include rotating, flipping, scaling, cropping, etc. [22]. Because traditional data augment methods easily cause over fitting and under fitting, we present a GAN-based method for Martian rock image data generation, which can generate a large amount of true, diverse Mars images just by training with a few Martian rock images.

**StyleGAN-Based Images Generating.** Generative Adversarial Networks (GAN) [23] are proposed to generate realistic-looking images from random latent code using neural networks. It consists of two sub-networks: Generator and discriminator. During the training, the generator is used to synthesize images to fool the discriminator; meanwhile, the discriminator tries to distinguish real and fake images. Generally, the generator and the discriminator are trained simultaneously through competing with each other. In this paper, we define an input random latent z∈Z, where Z denotes the space distribution of the latent code. A generated fake image is defined as G(z), and the space distribution of fake images is G(Z). The distribution of real image *x* is X. The discriminant results for real images and fake images are D(x) and D(G(z)), respectively. We train the discriminator *D* to maximize the probability of assigning the correct label to both real and fake images, and we simultaneously train the generator *G* to minimize log(1−D(G(z)). In other words, *D* and *G* play the following two-player min–max game with value function V(G,D):

(1) $$\begin{array}{c} \min_{G}\;\max_{D}\;E_{x∼X}\left[\log D\left(x\right)\right]+E_{z∼Z}\left[\log\left(1-D\left(G\left(z\right)\right)\right)\right] \end{array}$$

In order to further improve the ability to fit the image distribution, [24] scale GAN using CNN architectures. GAN used to be considered to have an unstable structure, [25] demonstrate that the traditional GAN uses the Jensen–Shannon divergence (JSD) between G(Z) and X as the loss to control the optimization process, but if the two distributions have supports that are disjoint or lie on low dimensional manifolds, the JSD will be constant, causing the gradient to disappear. So [26,27] use Wasserstein distance, which can always be used to quantify the difference between two distributions, instead of JSD as the loss for training GAN, and this method has been proved to improve the stability of training. Reference [28] proposes a training methodology for GAN where training starts with low-resolution images, and then progressively the resolution is increased by adding layers to the networks.This both speeds the training up and greatly stabilizes it, allowing the generator to produce high quality images. Spectral normalization, self-attention mechanism and largely enlarged network model are all applied to GAN and can greatly improve the quality of the generated images [29,30,31]. Despite these modifications improve the resolution and quality of the images produced by GAN rapidly while the generator continue to operate as a black box, so we still don’t understand many aspects of the image generation process. A style-based generator is proposed with the goal that image synthesis can be controlled by modifying the latent [18]. In recent years, there have been some efforts to use GAN for data enhancement [32,33], but none of them pay attention to the diversity of the generated images. With the style transfer technology of style-based generator, we can freely mix the styles of the generated images to synthesize a great variety of images.

**Figure 7:**
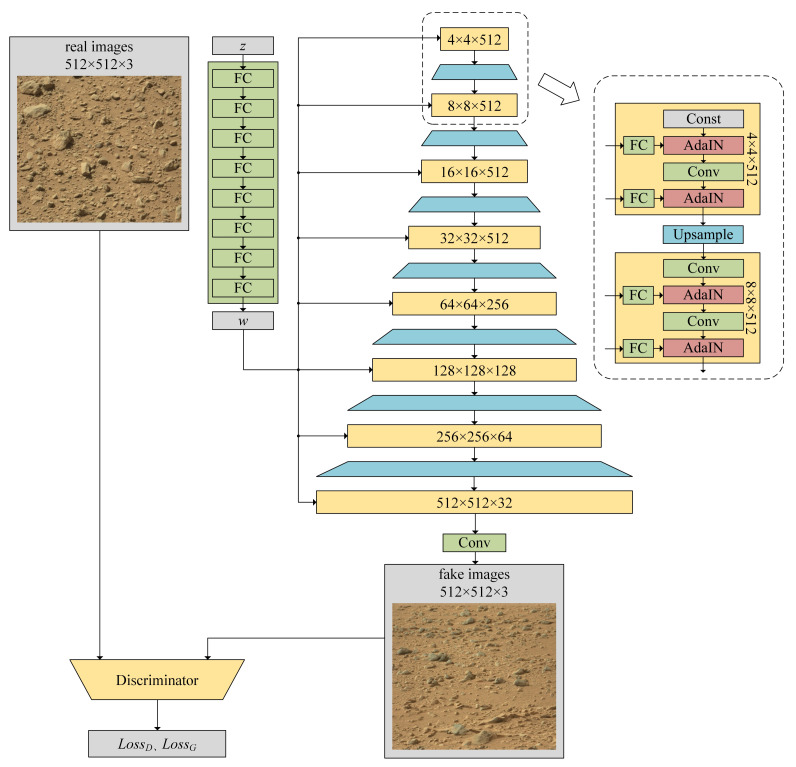
The structure of the GAN, which is being trained to generate Martian rock images. Latent *z* is mapped to *w* through an eight-layer fully connected network, and then *w* is used to control AdaIN operations after each convolution layer. After seven instances of upsampling, the size of the feature map grows from 4 × 4 to 512 × 512. The calculation process of each scale contains two convolution kernels and two AdaIN calculations, except for a 4 × 4 scale which includes one convolution kernel, two AdaIN calculations and one constant. A 512 × 512 × 32 feature map is converted to RGB using a separate 1 × 1 convolution. $loss_{D}$ and $loss_{G}$ are calculated from the output of the discriminator and are used to update the network weights of the discriminator and the generator, respectively.

The style-based generator uses a Multi-layer Perceptron (MLP) *f* to map *z* to w∈W, where W denotes a new space distribution of latent code *w*. The mapping procedure w=f(z) is conducive to disentanglement, and its goal is a latent space that consists of linear sub-spaces, each of which controls one factor of variation, and a variation factor corresponds to a visual feature of the generated image. In our settings, the sampling density of latent space W is not fixed because the mapping f(z) could be trained. So the mapping $f(z)\overset{}{\to }G(f(z))$ is to be more linear than $z\overset{}{\to }G(z)$. Latent code *w* is specialized to styles $y=(y_{s},y_{b})$ by learned affine transformations, where *y* are used to control adaptive instance normalization (AdaIN) [34], an operation that enables *y* to influence generated images. There are a total of 16 feature maps in our network that need to be normalized by AdaIN, the AdaIN operation of layer *i* can be formulated as follows:

(2) $$\begin{array}{c} \mathrm{AdaIN}\left(x_{i},y\right)=y_{s,i}\frac{x_{i}-\mu \left(x_{i}\right)}{\sigma (x_{i})}+y_{b,i} \end{array}$$

where feature map $x_{i}$ is first normalized by its mean $\mu (x_{i})$ and standard deviation $\sigma (x_{i})$, and then scaled by $y_{s,i}$ and biased by $y_{b,i}$ using the corresponding scalar components from style *y*. Specifically, we use a style-based generator to generate 512 × 512 pixel images, and then resize these images to 560 × 500 pixels. The structure of the GAN is shown in Figure 7, which is being trained to generate Martian rock images. Training of the generator is progressively growing by the up-sampling step, which is a methodology that starts with low-resolution images and then progressively increases the resolution by adding layers to the networks. This incremental nature allows training to first discover the large-scale structure of the image distribution and then pay attention to increasingly finer scale details, rather than learning all the scales at once. A discriminator outputs the judgment scores of real and fake images, respectively, which are used to calculate the loss.

**Figure 8:**
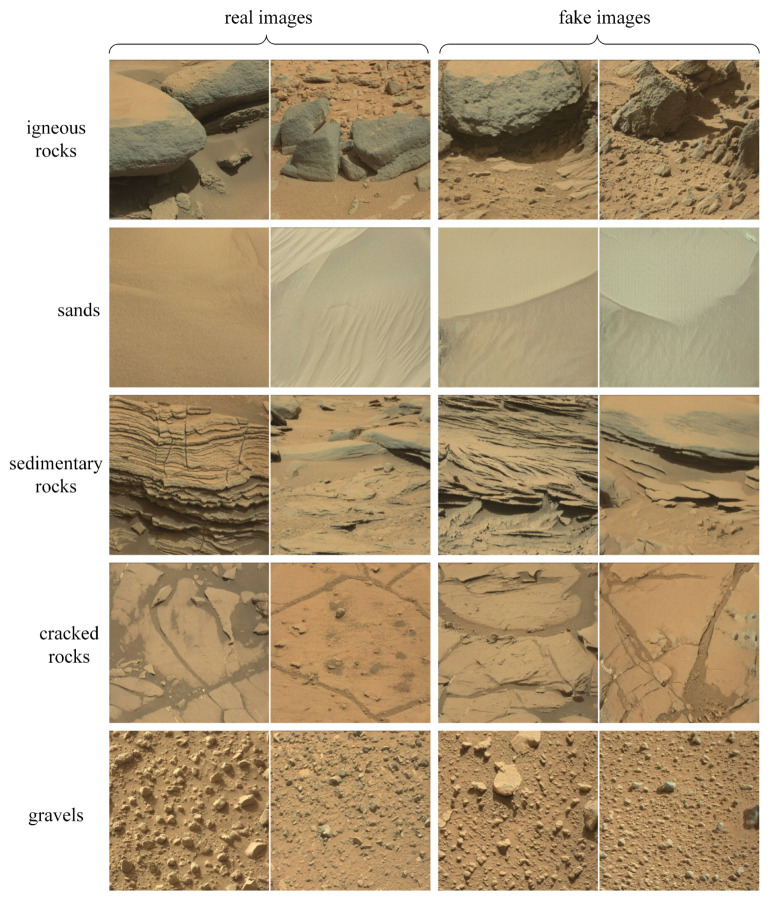
The comparison between the real images and the generated Mars images. Two real images and two generated Mars images were extracted from each subset for comparison and visualization, and further showing the effectiveness of the image generation method.

Moreover, we divide the Martian rock images in the training set into five categories and generate each type of image with the trained generator. The comparison between the real images and the generated fake images are shown in Figure 8. By comparing the real images and the generated images of the five rock categories, it can be determined that the generator can generate realistic Mars images.

**Image synthesizing based on style mixing.** The trained generator can learn the distribution of feature layers at different levels separately. This mechanism can be used to control image synthesis from texture and shape which are key features of rock. In the network of the generator, the layers that were trained in the early stages control the large-scale structure of the generated images, but the layers that were trained in the later stages control the fine-scale details of the generated images.

**Figure 9:**
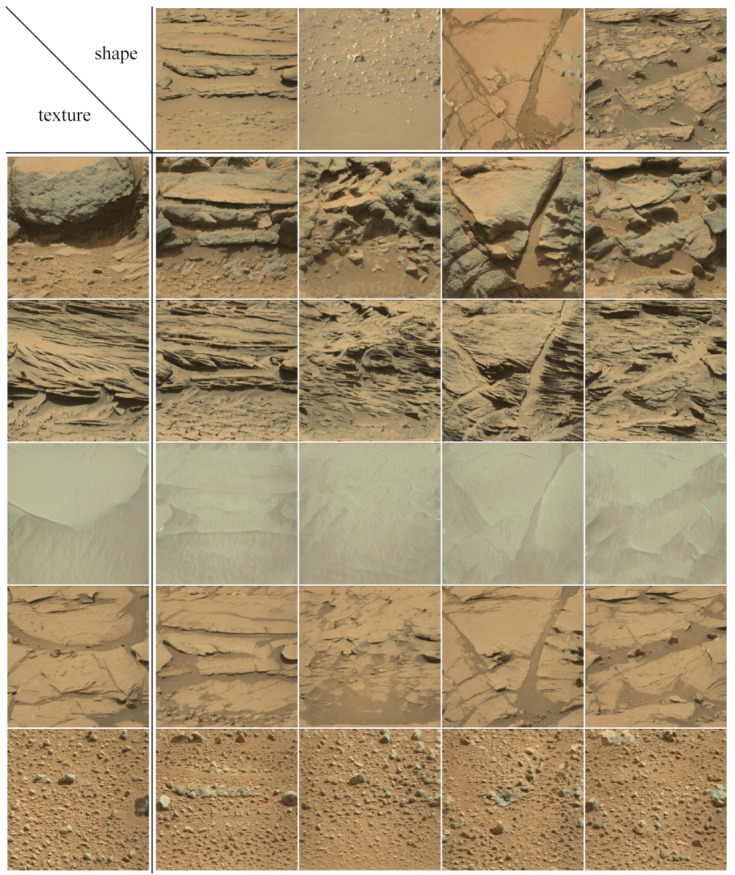
Examples of Martian rock images synthesised by style mixing. The mixed results of five rock textures and four spatial structures are exhibited. It can be seen that after style mixing, the texture of various kinds of rock images in the “texture” list has not changed, but their shape and spatial structure become similar to rock images in the “shape” list.

In the process of synthesising Mars images, we use an eight-layer MLP to map two latent codes $z_{1}$ and $z_{2}$ to $w_{1}$ and $w_{2}$, respectively. Latent codes $w_{1}$ is used to influence the shape of the generated images by substituting it into the AdaIN calculations where the spatial resolution is coarse (4 × 4 to 32 × 32). Latent codes $w_{2}$ is used to influence texture of the generated images by substituting it into the AdaIN calculations where the spatial resolution is fine (64 × 64 to 512 × 512). With the style mixing technique, we can finely control the image generation for targeted dataset expansion; a few examples of synthesis images using this technique are shown in Figure 9.

In Figure 9, the five rock types correspond to five textures, so we select one generated image from each type and use its latent codes $w_{2}$ to affect the texture of the synthesized images. In addition, we select four generated images with different spatial structures and use the latent codes $w_{1}$ to affect the shape of the synthesized images. The results show that the control of image synthesis is in line with our expectation.

### 3.4. Evaluation Metric of the Generated Images

With the Martian rock images being generated, in this sub-section, we experimentally demonstrate that the fidelity of the generated fake images is close to the real images. Fréchet Inception Distance (FID) [35], also known as Wasserstein-2 distance [36], is chosen for the quantitative evaluation. FID is a principled and comprehensive metric, and has been shown to be more consistent with human evaluation in assessing the realism and variation of the generated samples [37]. Let $p_{f}$ denote the sample distribution of the generated images and $p_{r}$ denote the distribution of the samples from the real images. The Fréchet distance between the Gaussian with mean and covariance $\left(m_{f},C_{f}\right)$ obtained from $p_{f}$ and the Gaussian $\left(m_{r},C_{r}\right)$ obtained from $p_{r}$ is called FID, which is calculated by [38]:

(3) $$\begin{array}{c} \mathrm{FID}=\sqrt{∥m_{f}-m_{r}{∥}_{2}^{2}+T_{r}\left(C_{f}+C_{r}-2{\left(C_{f}C_{r}\right)}^{1/2}\right)} \end{array}$$

**Figure 10:**
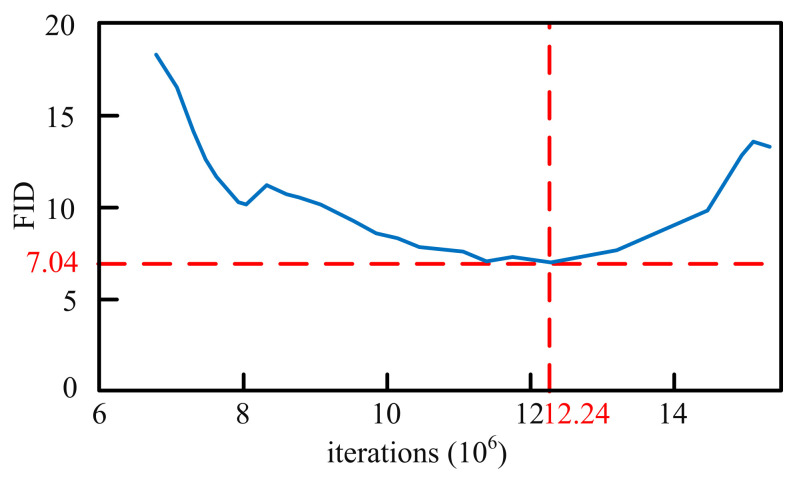
The FID-iterations curve of images generated by the trained model. The horizontal axis represents the number of iterations, and the vertical axis represents the FID between the distribution of generated images and the distribution of real images. It can be seen that when the number of iterations of model training reaches 12.24 million, FID reaches the minimum value of 7.04.

While evaluating the quality of generated images, in order to select the best trained model, we sample trained models with different iterations and calculate their FID. The FID-iterations curve is shown in Figure 10. Through comparative analysis, we select the images generated by the model after 12.24 million times of training to expand the dataset, where FID the minimum value of 7.04.

## 4. GMSRI Applications

In this section, we will introduce an application of our GMSRI, i.e., image super-resolution. Specifically, we train the kernelGAN [39] by using our GMSRI dataset, and compare the achieved performance with some classic methods including EDSR [40], ESRGAN [41] and ZSSR [42]. The evaluation indicators peak signal-to-noise ratio (PSNR) and structural similarity (SSIM) are calculated in Table 1.

As shown in Table 1, training the KernelGAN [39] by using our GMSRI dataset can achieve satisfactory results, i.e., 26.42/0.72 and 25.74/0.628 in PSNR/SSIM with ×2 and ×4 scales, respectively, which is a baseline for comparison by other researcher in the future.

Furthermore, our GMSRI dataset can also be used in other applications for Mars exploration, e.g., Mars object detection, Mars terrain segmentation, etc. We hope our GMSRI dataset can be widely used in the research of Mars exploration.

## 5. Conclusions

In this work, we present GMSRI, a texture-based Martian surface rock image dataset that consists of images selected from mars32K and images generated by a style-based generator. Comparing with the existing Martian surface image dataset, GMSRI organizes images in a hierarchy and provides a large number of images that have been classified from the texture and spatial structure of Martian rocks. The goal of GMSRI is to tackle the problem that Mars rock images are difficult to obtain and therefore there is a lack of training samples. In order to prove that GMSRI has achieved the purpose for which it was established, we verify that GMSRI can improve the performance of the Mars rock image processing algorithm through an experiment of the training image super-resolution network. We hope that GMSRI can promote the exploration and standardized evaluation of advanced techniques for Mars rover Visual task execution systems in the research community.

## Figures and Tables

**Table 1:** Quantitative results for super-resolution on GMSRI. We show the performance of KernelGAN [39], EDSR [40], ESRGAN [41], ZSSR [42] in PSNR/SSIM with ×2 and ×4 scales.

Method/Scale	×2	×4
EDSR [40]	26.03/0.49	23.06/0.42
ESRGAN [41]	23.26/0.58	20.22/0.51
ZSSR [42]	25.52/0.62	23.58/0.58
**KernelGAN** [39]	26.24/0.72	25.74/0.628

## Data Availability

The dataset and source code will be released at https://github.com/Sieann96/GMSRI.
